# Efficient Generation of Corticofugal Projection Neurons from Human Embryonic Stem Cells

**DOI:** 10.1038/srep28572

**Published:** 2016-06-27

**Authors:** Xiaoqing Zhu, Zongyong Ai, Xintian Hu, Tianqing Li

**Affiliations:** 1 School of Life Sciences, University of Science and Technology of China, Hefei, Anhui 230000, PR China; 2 Yunnan Key Laboratory of Primate Biomedical Research; Institute of Primate Translational Medicine, Kunming University of Science and Technology, Kunming, Yunnan 650500, China; 3 Kunming Ennovate Institute of Bioscience, Kunming, Yunnan 650500, China; 4 Key Laboratory of Animal Models and Human Disease Mechanisms of Chinese Academy of Sciences & Yunnan Province, Kunming Institute of Zoology, Kunming, Yunnan 650223, China

## Abstract

Efforts to study development and function of corticofugal projection neurons (CfuPNs) in the human cerebral cortex for health and disease have been limited by the unavailability of highly enriched CfuPNs. Here, we develop a robust, two-step process for generating CfuPNs from human embryonic stem cells (hESCs): directed induction of neuroepithelial stem cells (NESCs) from hESCs and efficient differentiation of NESCs to about 80% of CfuPNs. NESCs or a NESC faithfully maintain unlimitedly self-renewal and self-organized abilities to develop into miniature neural tube-like structures. NESCs retain a stable propensity toward neuronal differentiation over culture as fate-restricted progenitors of CfuPNs and interneurons. When grafted into mouse brains, NESCs successfully integrate into the host brains, differentiate into CfuPNs and effectively reestablish specific patterns of subcortical projections and synapse structures. Efficient generation of CfuPNs *in vitro* and *in vivo* will facilitate human cortex development and offer sufficient CfuPNs for cell therapy.

The cerebral cortex is one of the most complicated tissues in our brain, and its impairments result in neurodevelopmental and neurodegenerative disorders, such as sensory, emotional, cognitive and motor function defaults^1^,^2^. The cortex is comprised of six horizontal layers, which contain two unique morphological neurons: glutamatergic excitatory pyramidal projection neurons and GABAergic inhibitory interneurons^3^,^4^,^5^. Pyramidal projection neurons, also called cortical projection neurons (CPNs), account for approximately 80% of all cortical neurons and serve as both the sole output from and the largest input system to the cortex. Accordingly to their distinct locations and synaptic connectivity patterns, CPNs are classified as corticofugal projection neurons (CfuPNs), that reside in layers 5 and 6 (deep layer), and intracortical projection neurons, that are found mostly within layers 2/3 (upper layer)^6^. CfuPNs send their axons to subcortical targets such as the thalamus and subcerebral targets such as the midbrain and spinal cord, and control discrete voluntary movements^7^.

Human pluripotent stem cells including embryonic stem cells (hESCs) and induced pluripotent stem cells (iPSCs) have emerged as promising cells to model brain development and study disease, and hold remarkable values for regeneration medicine^8^,^9^,^10^. At present, some studies have successfully induced the generation of mature cortical neurons from ESCs by using a few small molecular compounds^2^,^3^,^11^,^12^,^13^,^14^,^15^,^16^,^17^,^18^,^19^ or by using 3D suspension culture specific for promoting human corticogenesis with respect to layer-specific neurogenesis and regional specification^3^,^20^,^21^,^22^. Although these systems can model cortical development well, their differentiated cells are a mixed cell population including upper layer and deep layer cortical neurons. It is obscure whether highly enriched CfuPNs have been generated from hESCs. In addition, these cortical progenitors described by these systems are difficult to provide sufficient cell population with stable characteristics for cell differentiation, disease studies and cell therapy due to limited proliferation abilities^23^. Therefore, developing a simple culture system to obtain stem cells with the ability to efficiently generate CfuPN in a stable, controlled and conserved manner will provide the advantage for exploring molecular mechanisms underlying CfuPN differentiation and cell therapies of cortex diseases.

Because of its well-known poor/limited plasticity, the adult cortex has a poor ability to self-repair in many diseases, such as stroke, epilepsy, cortex injury and neurodegeneration, by generating new cells^24^. Therefore, transplantation of NPCs/NSCs becomes the key strategy for cell replacement in the central neural system. Some studies have demonstrated the possibility of cell transplantation for neural repair of cortical lesions^25^,^26^,^27^,^28^,^29^. Of note, a recent report showed that neurons with visual cortex identity, derived from mouse ESCs, were transplanted successfully following a lesion of the adult mouse visual cortex, and reestablished the damaged pathways including long range and reciprocal axonal projections and synaptic connections with targets of the damaged cortex^30^. Although these studies open the possibility of cell transplantation for cortical repair, the limited expansion ability of these cells in the culture become obstacles for their future applications. Therefore, it is crucial to generate transplantable cortical stem cells with the ability to produce CfuPNs *in vivo* after long-term culture.

## Results

### Generation of neuroepithelial stem cells from human ESCs

**Figure 1:**
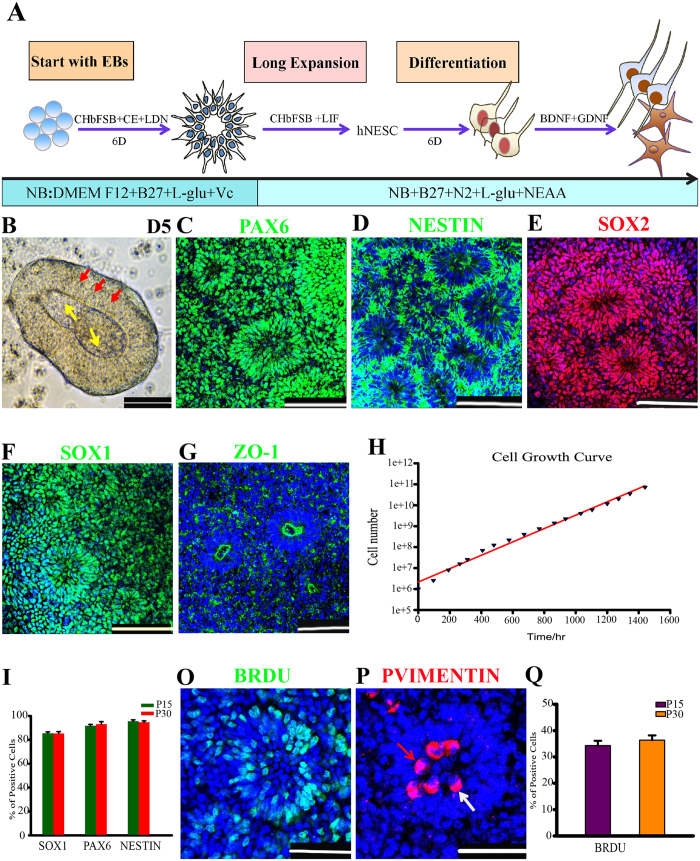
Generation of long-term self-renewal neuroepithelial stem cells. (**A**) Schematic of the differentiation protocol for generating neuroepithelial stem cells (NESCs). CH, CHIR99021; bF, bFGF; CE, Compound E; NB, Neurobasal medium. The drawings were drawn by the author Xiaoqing Zhu. (**B**) Neural bodies at pdD5 attached and formed a two-layer structure after cultured onto laminin-coated plates for two additional days in the NESC culture media, yellow arrows and red arrows indicate two different layers, respectively. (**C–G**) Long-term cultured NESCs were organized into neural tube-like structures expressing neuroepithelial cell markers: PAX6, NESTIN, SOX2, SOX1 and ZO-1. (**H**) The growth curve of NESCs, displaying the prospect of exponential growth over serial passages. (**I**) Quantifications of PAX6, SOX1 and NESTIN positive cells in P15 (Passage 15) and P30 NESCs. Data expressed as mean ± s.d (n = 3) (P > 0.05 as Student’s *t*-test). (**O**) BrdU labeling identified most of the S-phase cells to be located on the basal surface of a neural tube (NT). (**P**) Mitotic marker phospho-VIMENTIN (p-VIMENTIN) positive cells were mainly distributed at the apical surface of a NT. The white arrow indicates symmetrical horizontal division orientation, and red indicates vertical division orientation. (**Q**) Quantification of BrdU incorporating cells in P15 and P30. Data expressed as mean ± s.d (n = 3) (P > 0.05 as Student’s *t*-test). Scale bars: 100 μm. Blue: DAPI, nuclear staining.

The initial cortical progenitors originate from neuroepithelial stem cells (NESCs)^23^,^31^, which hold strong proliferative ability, display rosette architectural feature, and are positive for PAX6^21^,^32^,^33^. Previous studies showed that inhibitions of TGF/Nodal, Notch and BMP4 signaling pathways or activation of Wnt signaling pathway have the abilities to support the generation of cortical progenitors from PSCs^2^,^11^,^13^,^14^,^15^,^19^,^21^,^32^,^33^,^34^,^35^. To test whether NESCs could be quickly and effectively obtained, hESCs were induced differentiation by the combined usage of inhibitors of TGF/Nodal, Notch and BMP4 pathways supplemented with the agonist of Wnt pathway^35^ (Fig. 1A). Using this approach, hESCs were rapidly converted into neuroepithelium-like structures within five days in suspension culture (Fig. 1A). NESTIN and ZO-1 immunofluorescence indicated that these structures formed a typical two-layer neural body (NB) structure (Fig. S1A,B) after transferred and cultured on laminin-coated plates for two days (Fig. 1B), which were reported to be an important structure for recapitulating telencephalon development *in vitro*^3^,^20^,^21^. We also found that highly enriched NESCs were established from these two-layered NBs. In contrast, the usage of NBs without two-layer structure resulted in the generation of heterogeneous NESCs (data not shown). NBs were subjected to dissociation and re-plating/passaging, which could be further passaged for at least 30 passages in CHbFSB + LIF media^35^,^36^ (Fig. 1I). Upon re-plating, these cells were organized into polarized miniature neural tube (NT)-structure, uniformly expressing PAX6, NESTIN, SOX2 and SOX1 (Fig. 1C–F). Clustering ZO-1 marking the luminal side implied that these cells were polarized NESCs (Fig. 1G).

These cells displayed exponential growth over serial passages and resulted in a 2 × 10^5^ fold increase within two-months without losing obvious proliferative capacity (Fig. 1H), implying that large numbers of cells can be rapidly provided for future applications in the short term. PAX6, NESTIN and SOX1 expressions were stably sustained over extensive propagation (Fig. 1I). In addition, most of BrdU labeled S-phase nuclei were found to be on the basal surface (Fig. 1O), whereas phospho-VIMENTIN labeled dividing cells were located on the apical surface (Fig. 1P), similar to the cell division of interkinetic nuclear migration (IKNM) found in developing NTs *in vivo*^37^. BrdU incorporation also revealed that early- (P15) and late- (P30) passaged cells maintained similar proliferation abilities (Fig. 1Q). Furthermore, these NESCs were negative for GFAP and BLBP (two radial glial makers), and Tbr2 (an intermediate progenitor markers) (Fig. S1C–S1D), whereas removal of CHIR99021 alone from CHbFSB + LIF media resulted in the loss of NT formation and subsequent transition of “NESCs-TO-radial glial progenitor cell (RGPC)” within 14 days (Fig. S1E), consistent with our previous report in monkey NESCs^35^. Together, these properties indicated that these cells displayed typical and stable neuroepithelial characteristics of *in vivo* NTs within long-term culture.

### hESCs-derived neuroepithelial stem cells gave rise to mature neurons

**Figure 2:**
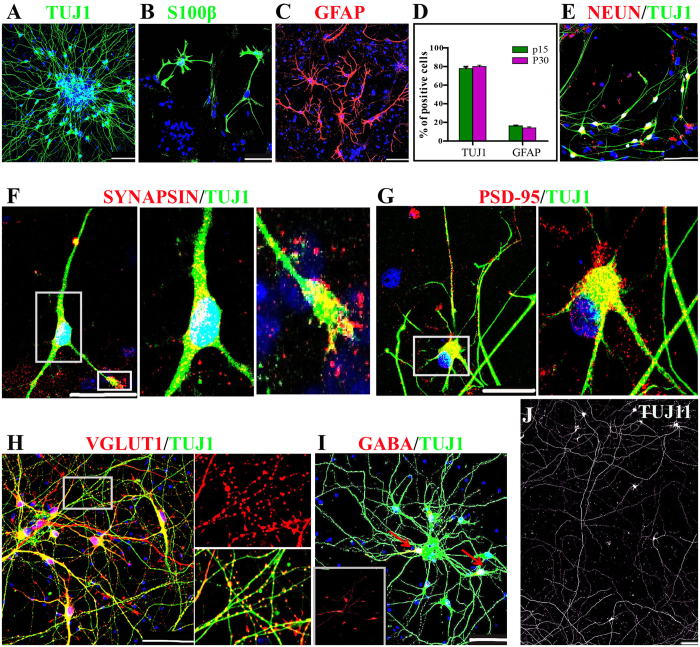
Neuroepithelial stem cells (NESCs) give rise to physiological mature neurons. (**A–C**) NESCs differentiated into TUJ1^+^ neurons, S100β^+^ (**B**) and GFAP^+^ (**C**) astrocytes at 28 days post-differentiation (pdD28). (**D**) Quantifications of TUJ1 and GFAP positive cells derived from P15 and P30 NESCs at pdD28 demonstrate that NESCs maintained stable neurogenic abilities after extensive passaging. Data expressed as mean ± s.d (n = 3) (P > 0.05). (**E**) NESCs developed into NeuN^+^ neurons at pdD40. (**F**,**G**) NESCs-derived neurons expressed the pre-synaptic protein, SYNAPSIN I at pdD46, and the post-synapse marker, PSD-95 at pdD71 in a punctate pattern. (**H**,**I**) NESCs differentiated into glutamergic and GABAergic positive neurons at pdD46. (**J**) The axons regenerated from NESCs could extend up to 2 cm in the length at pdD46. Scale bars: (**F,G**), 50 μm, the others 100 μm. Blue: DAPI, nuclear staining.

To examine the neurogenic and gliosis potentials, hESCs-derived NESCs were induced differentiation after removal of bFGF, LIF, CHIR99021 and SB431542. Four weeks later, the inmunofluorescence staining showed that NESCs retained a stable propensity toward TUJ1^+^ neuron and S100-β^+^ GFAP^+^ astrocyte differentiation over extensive passages (Fig. 2A–C). To further exclude the presence of radial glial cells, we also checked Pax6 expression and did not detect any Pax6 positive cell in differentiated cells (data not shown). Quantification data showed that NESCs at passage 15 and 30 gave rise to 77.6 ± 5.0% and 79.8 ± 3.5% of TUJ1^+^ neurons, as well as 17.51 ± 2.0% and 16.32 ± 4.3% of GFAP^+^ astrocytes, respectively (Fig. 2D). Synaptogenesis is a critical step of neural circuit formation. To test whether these NESCs-derived neurons could form physical synapses *in vitro*, differentiated neurons at Day 13 post-differentiation (pdD13) were co-cultured with mouse embryonic cortical astrocytes. More than 90% of the differentiated neurons were found to express NeuN (Fig. 2E), and form synapse structures expressing pre-synaptic SYNAPSIN I and post-synaptic PSD-95 protein in their axons in a punctate pattern after 33 days and 58 days of co-culture, respectively (Fig. 2F,G). Our experiments also showed that most of neurons derived from NESCs outgrew an axon with the length of more than 2 mm at pdD46 (Fig. 2J). Subtype identifications showed that differentiated neurons were composed of 76 ± 3.1% vGLUT1 positive excitatory neurons and 21.5 ± 8.9% GABA inhibitory neurons (Fig. 2H,I), whereas other subtype neurons, such as tyrosine hydroxylase (TH), 5-HT and ChAT positive neurons, were not detected (Data not shown).

### NESCs expressed cortical deep layer markers

Because of lack of production of TH, 5-HT and ChAT neurons, we hypothesized that the fate of NESCs may be restricted to telencephalon. To verify this hypothesis, telencephalic markers expressions were checked. As expected, RT-PCR and immounstaining data showed NESCs express telencephalic markers, such as *PAX6*, *OTX1*, *FOXG1* and *FEZF2* transcription factors, but not for ventral telencephalic marker NKX2.2 (Fig. S1F,G). Recent work suggests that cortex may contain intrinsically fate-restricted progenitors marked by expression of Cux2^38^. To test whether NESCs are fate-restricted progenitors, the expression of layer specific markers was also analyzed. Surprisingly, NESCs expressed FOXP2 (a cortical deep layer marker), which is aslo found to express in some cortical RGPCs of mouse E12.5 brain^39^, along with few SATB2^+^ (a marker of callosal neurons from layers V or upper layer neurons) cells, but not for TBR1 (a marker of cortical projection neurons), while the expression of either BRN2 or Cux1 (two cortical upper layer markers) was not detected (Fig. S1H,I).

### NESCs differentiated into highly enriched CfuPNs along with a few interneurons

**Figure 3:**
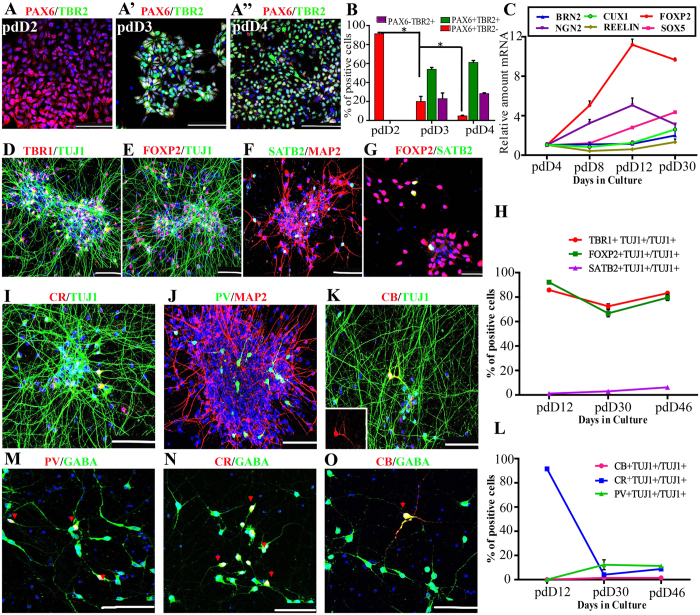
Neuroepithelial stem cells (NESCs) give rise to highly enriched CfuPNs along with a few interneurons. (A–A”) NESCs underwent the progression of Pax6^+^ Tbr2^−^ NESCs at pdD2 (**A**), Pax6^+^ Tbr2^+^ RGPCs at pdD3 (A’) and Pax6^−^Tbr2^+^ intermediate progenitors at pdD4 (A”) before neurogenesis after spontaneous differentiation, respectively. (**B**) Quantification of TBR2 and PAX6 positive cells during neurogenesis. Data expressed as mean ± s.d (n = 3) (*P < 0.05). (**C**) qRT-PCR data show the expression dynamics of apical cortical progenitors’ marker *NGN2* and transcription factors specific for corticogenesis. (**D,E**) NESCs give rise to TBR1^+^ and FOXP2^+^ CfuPNs at pdD46. (**F**,**G**) NESCs give rise to a few SATB2^+^ FOXP2^+^ layer V callosal neurons at pdD28 (one subtype of CfuPNs). (**H**) Quantification of projection neurons corresponding to cortical deep layers during the differentiation course indicated by immunofluorescence staining, respectively. Data expressed as mean ± s.d. (**I–K**) Three subtypes of interneurons generated from NESCs at pdD46. CR: CALRETININ; PV: PARVALBUMIN; CB: CALBINDIN. (**L**) Quantification of three subtypes of interneurons during corticogenesis, respectively. Data expressed as mean ± s.d. (**M–O**) Some representative PV- (**M**), CR-(**N**), CB-(**O**) with GABA double positive inhibitory interneurons in the differentiated cells from NESCs at pdD35. Red arrows indicate double positive neurons. Scale bars: 10 μm. Blue: DAPI, nuclear stain.

To evaluate differentiation potential of NESCs, differentiated neurons were further identified. Neurons from different layers are generated at distinct time points, where the earliest-born neurons generated in the cortex were Cajal–Retzius neurons, followed by deep cortical layer CfuPNs, then by upper layers IV, and lastly layers II/III intracortical projection neurons^23^,^38^,^40^,^41^. First, whether NESCs underwent RGPC and intermediate progenitors (IPs) before neurogenesis was examined^42^. During the course of differentiation, 91.41 ± 2.47% of cells were PAX6^+^ TBR2^−^ NESCs at pdD2, which subsequently gave rise to 53.83 ± 3.54% of PAX6^+^ TBR2^+^ RGPCs at pdD3 and 28.32 ± 1.39% of PAX6^−^TBR2^+^ IPs at pdD4 (Fig. 3A,A”,3B). In contrast, PAX6^+^ TBR2^−^ NESCs dramatically declined to 4.76 ± 1.25% at pdD4. Consistent with the result, the expression of *NGN2*, an apical cortical progenitor markers, increased at a gradient level within the first twelve days, as shown by quantitative RT-PCR (Fig. 3C).

With these encouraging results, the expression of a large set of layer-specific markers corresponding to the major subtypes of cortical neurons throughout the *in vitro* neurogenesis process was examined. The results of qRT-PCR showed that compared with pdD4, some transcription factors, such as *SOX5* and *FOXP2*, specific for CfuPNs were upregulated over differentiation (Fig. 3C), whereas the *BRN2* and *CUX1*, the upper layer (II–IV) marker, and *REELIN*, a maker of MZ Cajal-Retzius neurons, almost remained unchanged or a slight increase (Fig. 3C). Consistent with qRT-PCR, immounstaining showed that TBR1^+^, FOXP2^+^ and SATB2^+^ neurons significantly increased in differentiated cells (Fig. 3D–G). Quantifications showed that 81 ± 1.35% and 77.05 ± 1.39% of differentiated neurons were TBR1^+^ and FOXP2^+^ CfuPNs at pdD46, respectively (Fig. 3D,E,H). Interestingly, the presence of 4.69 ± 2.46% of SATB2^+^ neurons was also observed (Fig. 3F,H). Due to SATB2 corresponding to callosal neurons from layers V and upper layer neurons, the FOXP2 expression in SATB2^+^ neurons was checked and the result showed that all SATB2^+^ cells were positive for FOXP2, indicating that these SATB2^+^ cells were layer V callosal neurons (Fig. 3G). In contrast, Brn2 or Cux1 positive intracortical projection neurons were not detected in the differentiated cells even up to pdD70 (Fig. S1J), in which the period is enough to produce upper layer neurons based on previous reports^18^,^20^,^21^,^22^,^33^. Taken together, our data showed that NESCs can specifically gave rise to highly enriched CfuPNs.

In addition, it was also noted that about 20% of differentiated neurons were not pyramidal neurons. In the cortex, interneurons constitute about 20% of the total number of cortical neurons^5^,^43^. According to the expression of calcium binding proteins, cortical interneurons are composed of three main subtypes, such as Parvalbumin, Calbindin and Calretinin^5^,^17^,^43^,^44^. The characterization demonstrated the presence of Calretinin^+^ (7.5 ± 1.3%), Parvalbumin^+^ (10.4 ± 1.6%) and Calbindin^+^ (1.2 ± 0.9%) neurons in differentiated cells, respectively (Fig. 3I–L). Furthermore, most of Calretinin-, Parvalbumin- and Calbindin- interneurons co-expressed GABA (Fig. 3M–O), confirming their identities of inhibitory interneurons. Considering that Somatostatin^+^ neurons are one of the major subtypes of GABAerigic neurons in corticogenesis, we checked their production. However, we failed to observe presence of any Somatostatin^+^ neurons in differentiated cells at pdD29 (Fig. S1K).

### A NESC self-organized into NTs and differentiated into CfuPNs and interneuron

**Figure 4:**
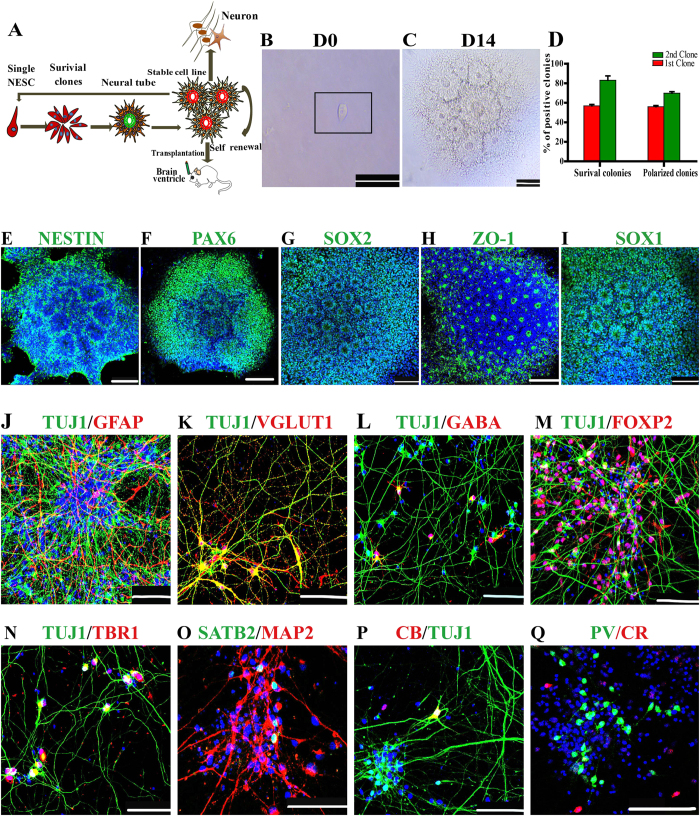
Single neuroepithelial stem cell (NESC) can self-organize into miniature neural tubes (NTs) and differentiate into CfuPNs and interneurons. (**A**) Schematic of the protocol for generating miniature NTs from a single neuroepithelial cell. The drawings were drawn by the author Xiaoqing Zhu.(**B**) A NESC seeded on one well of 96-well plate. (**C**) A NESC self-organized into polarized NTs on day 14. (**D**) Quantification of the percentages of survival colonies and polarized colonies derived from single human NESCs versus the number of inoculated single cells at Day 14 during continual colony assays. Data expressed as mean ± s.d (n = 4). (**E**–**I**) The polarized NTs express NESC markers, such as NESTIN, PAX6, SOX2, ZO-1 and SOX1. (**J**) Single NESC-derived progenies differentiated into TUJ1^+^ neurons and GFAP^+^ astrocytes at pdD26. (**K**,**L**) Single NESC-derived progenies differentiated into glutamergic (vGLUT1^+^) and GABAergic (GABA^+^) positive neurons at pdD35. (**M–Q**) Single NESC-derived cells differentiated into FOXP2^+^, TBR1^+^ and SATB2^+^ CfuPNs at (**M–O**) and CB^+^, CR^+^, PV^+^ interneurons (**P,Q**) at pdD40. Scale bars: 100 μm. Blue: DAPI, nuclear staining.

Given that NESCs in the miniature NTs have strong proliferation and neurogenesis ability, whether single NESCs can undergo serial clonal expansion to self-organize into NTs was explored (Fig. 4A). At one day after seeding in 96 well plates, 56.8 ± 2.4% of single seeded NESCs survived and exhibited stable proliferation with a highly homogenous morphology (Fig. 4B–D). 55.9 ± 2% of seeded cells formed rosette-structure with a lumen at day 12 and eventually generated NT structure at day 14 (Fig. 4C,D). These NT colonies uniformly expressed PAX6, NESTIN, ZO-1, SOX2 and SOX1 (Fig. 4E–I). Furthermore, these single NESC-derived polarized colonies (100%, 28/28) maintained long-term self-renewal and generated stable cell lines. To further confirm their long-term self-renewal abilities, the abilities of single NESC-derived NT colonies to generate secondary colonies were evaluated by using the same protocol above. The result showed that 83.6 ± 6.3% and 69.8 ± 2.3% of single seeded cells survived and generated secondary NT colonies, respectively (Fig. 4D). The proportions were higher than those of the first NT colony formation (Fig. 4D), implying that single NESC-derived NT colonies displayed more homogeneous phenotypes.

To address differentiation potential of a NESC, single NESC-derived progenies were induced differentiation. As expected, single cell-expanded progenies gave rise to TUJ1^+^ neurons and GFAP^+^ astrocytes (Fig. 4J). Further characterizations showed differentiated neurons were Glutamatergic and GABAergic neurons similar to parental NESCs (Fig. 4K,L). Subtype identification demonstrated that these differentiated neurons contained TBR1^+^, SATB2^+^ and FOXP2^+^ CfuPNs (Fig. 4M–O), as well as three subtypes of interneurons, such as Calretinin-, Parvalbumin- and Calbindin- inhibitory interneurons (Fig. 4P,Q). Consistent with parental NESCs, Cux1 or Brn2 positive intracortical projection neurons was not observed (data not shown). Together, these data showed that a NESC gave rise to CfuPNs, inhibitory interneurons and astrocytes.

### NESCs derived neurons integrated into mouse brain in a wide range after grafted

**Figure 5:**
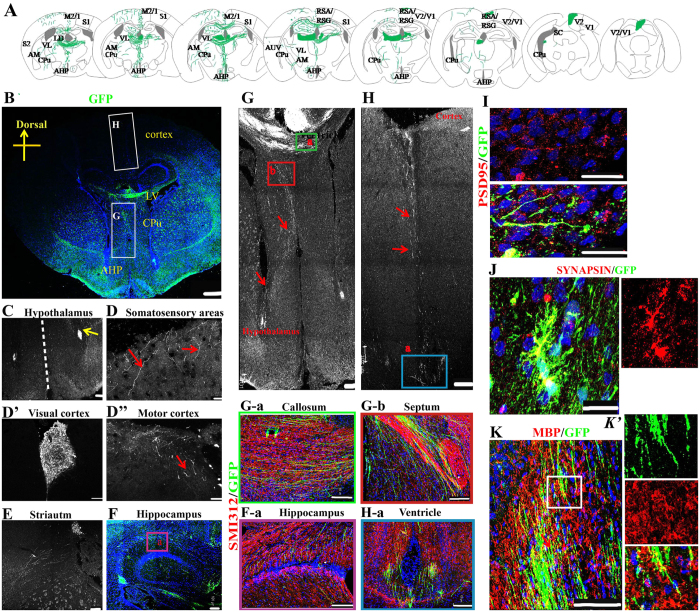
Single NESC-derived cells integrated well into brain and outgrew extensively axons after grafted into the ventricles of newborn mouse brain. (**A**) Camera lucida drawing shows an overview of integration and axonal projections of grafted cells one month after grafting. M2/1: motor cortex; S2/1; somatosensory cortex; V2/1: visual cortex; AHP: anterior hypothalamic area, posterior part; CPu: caudate putamen; RSA/RSG: retrosplenial granular cortex; AM: anteromedial thalamic nucleus; VL: ventrolateral thalamic nucleus; LD: laterodorsal thalamic nucleus; SC: Superior Colliculus; AUV: anteroventral thalamic nucleus. The drawings were redrawn by the author Xiaoqing Zhu following Ira Espuny-Camacho *et al*.^32^. (**B**) Representative axon outgrowths derived from grafted neurons in the mouse brain one month post-transplantation. LV: Lateral ventricles. (**C–F**) Confocal images show grafted GFP cells regenerate extensive fibers distributed in various brain regions one month post-transplantation. Red arrows show GFP positive fibers, and yellow arrows show grafted GFP cells. (F-a) The magnification picture with axon marker SMI312 staining. (**G**) Grafted GFP axon fibers extend from white matter tract of the corpus callosum to the hypothalamus through caudate putamen and thalamic. (**H**) Grafted GFP neurons regenerate axons along the orientation from the out surface cortex to ventricle across the whole cerebral cortex. Red arrows show GFP positive Fibers in (**G,H**). (G-a,b, H-a) The magnification images of axon marker SMI312 staining in (**G**,**H**). (**I,J**) The outgrowth axons extensively co-express post-synaptic protein, PSD-95 (**I**), and pre-synaptic protein, SYNAPSIN I (**J**), suggestive of formation of synaptic contacts between the transplanted neurons and the host neurons. (**K**) MBP staining indicates that GFP fibers colabeled with MBP, suggestive of myelination. (*K*’) The magnification image of the box in (**K**). Scale bars: (**B**), 500 μm; (**I,J**), 25 μm; others:100 μm. Blue: DAPI, nuclear staining.

To test the potential values of these NESCs in clinical application after long-term culture, their survival and differentiation *in vivo* were evaluated. First, NESCs (Passage 22) were infected with retrovirus including EF1a promoter driving green fluorescent protein (GFP). At three days after infection, cells were performed clonal expansion and single cell-derived stable cell lines were obtained. One GFP^+^ clonal cell line was randomly used for subsequent transplantation analysis. 4 × 10^5^ cells were transplanted into a new born SCID mouse ventricle (n = 11). The survival and integration of grafted cells were examined at one month post-transplantation using stereological analysis on serial coronal sections. Strikingly, these NESCs integrated and distributed in the whole mouse brains (Fig. 5A,B). Most of cells were located on the white matter tract of the corpus callosum (Fig. 5B). Of note, some cells migrated out the graft site and moved to surrounding brain areas, such as hypothalamus, cortex, anterior hypothalamic area and caudate putamen (Fig. 5B,C). Importantly, the fibers of grafted neurons outgrew and reached cortex and subcortical regions, including somatosensory areas (Fig. 5D), visual cortex (Fig. 5D’) and motor cortex (Fig. 5D”). Some GFP-positive axons outgrew into the striatum (Fig. 5E) and the left hippocampus (Fig. 5F).

Next, GFP^+^ axonal projections of the grafted neurons were examined, and then compared with the endogenous pattern of cortical projections. By analyzing the intact brain slice, two axon outgrowth orientations in most of grafted animals were observed: one was from the white matter tract of the corpus callosum to the hypothalamus through caudate putamen and thalamic (Fig. 5G), a typical characteristics of *in vivo* CfuPNs^7^; the other was from the out surface cortex to ventricle across the whole cerebral cortex (Fig. 5H). SMI312 (a marker of axon) staining demonstrated that the orientations of axon outgrowth were same to that of endogenous neuron axons (Fig. 5F-a,G-a,b, 5H-a and Movie S1). Together, these data indicated that NESCs derived neurons successfully integrated into the host brain and extend extensive projections that were strikingly similar to native CfuPNs.

Two months post-transplantation, staining data showed that 39 ± 5.9% of GFP positive cells were positive for neuron marker TUJ1 (Fig. S2A,D), whereas 17 ± 6.5% of grafted cells expressed the astrocyte marker GFAP (Fig. S2B,D). Furthermore, grafted cells expressed the mature neuronal marker NeuN (Fig. S2C,C’). Although a few GFP-positive cells expressed Ki67, a marker of proliferating cells (Fig. S2E,E’), tumors or Ki67^+^ proliferating cells were not observed in brain at the fifth month post-grafts (Fig. S2F,F’), suggestive of possible safety in further clinical application. These outgrowth axons extensively expressed SYNAPSIN I and PSD-95 protein in a punctate pattern (Fig. 5I,J), suggestive of synapse formation. GFP fibers were also detected to be extensively colabeled with MBP (Fig. 5K,K’), suggestive of myelination.

### Graft cells differentiated into CfuPNs *in vivo*

**Figure 6:**
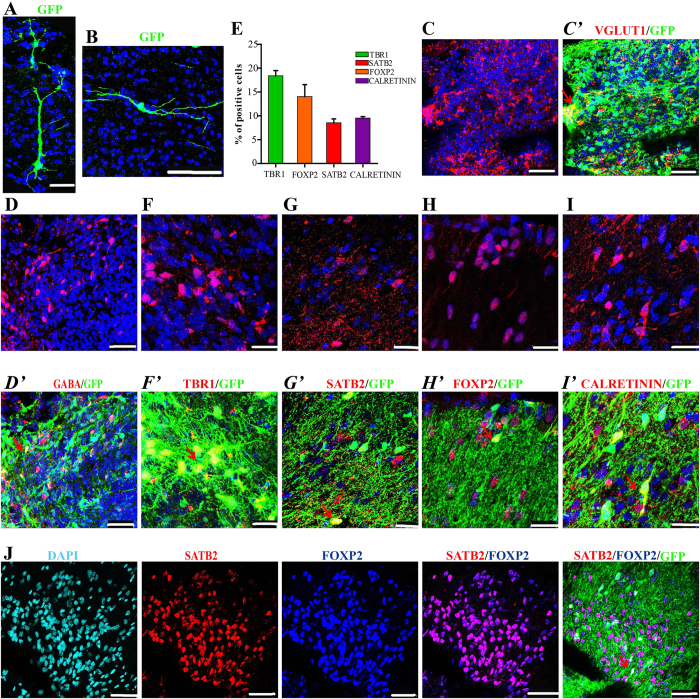
NESCs differentiated into CfuPNs and interneurons in the brains three months after grafted. (**A,B**) GFP^+^ neurons migrated out of graft sites displayed a pyramidal (**A**) or bipolar (**B**) morphology. (**C,D**) Grafted cells differentiated into vGLUT1 positive neurons (**C**) and GABA positive neurons (**D**). The two representative images are from two different regions of an implant. (**E**) Quantification of TBR1^+^, SATB2^+^, FOXP2^+^ and CALRETININ^+^ interneurons *in vivo* at three months after transplantation. Data expressed as mean ± s.d (n = 3). (**F–H**) The representative images of TBR1 (F,F’), SATB2 (G,G’) and FOXP2 (**H**,H’) CfuPNs derived from grafted cells. These representative images are from three different regions of an implant. (**I**) NESCs give rise to CALRETININ interneurons in *vivo.* (**J**) Immunofluorescence showed all SATB2^+^ neurons were positive for FOXP2. Red arrows indicate double positive Fibers. Scale bars: (**A**,**B**,**K)**, 100 μm; (**C,D,J**), 50 μm; F-I, 25 μm. Blue (except **J**): DAPI, nuclear staining.

Several reports showed that the cell fates of grafted NPCs are closely associated with the host brain environment and cell intrinsic properties^16^,^30^,^34^. To identify the potential that the grafted cells differentiated into CfuPNs and interneurons, the neurons’ morphologies *in vivo* were first analyzed. Within the cortex, CfuPNs exhibit a pyramidal morphology, characterized by the presence of one dendrite that is wider than others, whereas GABAergic interneurons show multipolar or bipolar morphologies^16^,^17^,^45^. Intriguingly, typical pyramidal and bipolar morphological neurons were observed in these migrated neurons (Fig. 6A,B and Movie S2).

Next, specific transcription factor antibodies with GFP were used to label subtypes of neurons in the brain sections. As expected, grafted cells in an implant were differentiated into vGLUT1 and GABAergic neurons (Fig. 6C,D), and 18.4 ± 1.8%, 14 ± 4.3% and 8.6 ± 1.4% of the GFP^+^ cells were TBR1^+^, FOXP2^+^ and SATB2^+^ neurons (Fig. 6E–H), respectively. Among them, all SATB2^+^ cells were positive for FOXP2 (Fig. 6J), suggestive of their identities of layer V callosal neurons. In contrast, no BRN2 or Cux1 upper-layer intracortical projection neurons were observed (Fig. S2G,H). Together, NESCs have the abilities to give rise to CfuPNs *in vivo* after graft. Additionally, the grafted NESCs were found to differentiate into 9.5 ± 0.5% of the Caleretin interneurons (Fig. 6I), but not Parvalbumin interneurons (Fig. S2I).

## Discussion

In summary, human NESCs are efficiently and robustly generated from ESCs in a defined media. NESCs are stably maintained in long-time culture and efficiently give rise to CfuPNs and inhibitory interneurons *in vitro* and *in vivo*, even at single cell level. The cells described in the study are found to (1) be polarized neuroepithelial cells, (2) self-organize into miniature NTs structure at a cellular level, (3) have robust expansion ability, (4) give rise to mature neurons and integrate well into brains, and (5) regenerate CfuPNs and inhibitory interneurons *in vitro* and *in vivo* at a cellular level. Based on these properties, NESCs probably are ideal cell models for stem cell therapy and disease studies.

The human cortex is composed of six-layer projection neurons and glia. During development, the projection neurons are generated from RGPCs and basal progenitors by an “inside-first, outside-last” manner^7^,^20^. CFuPNs that occupy the subplate (SP) and deep layers (L6 and L5) are generated first, followed by those in upper layers (L2–L4). Over the last decade, several methods have been developed from human ESCs and iPSCs for making a mixed population of cortical projection neurons including CfuPNs (deep layer) and intracortical projection neurons (upper layer)^12^,^17^,^33^,^34^. However, these systems only give rise to about 20–40% of deep layer neurons. Our study is the first time to show highly enriched (~80%) CfuPNs (deep layer neurons) could be generated from hESC-derived NESCs, providing an ideal cell mode to study CfuPN development.

During cortex development, an obscure question is whether neurons in different layers are generated by a common multipotent progenitor cell, separate fate-restricted progenitors, or a combination of both. Three decades of work involving cell transplantation, lineage tracing, transgenic reporter mice, chimeric mice, or *in vitro* culture of single RGCs demonstrate that cortical projection neuron subtype is sequentially determined by birthdate through progressive lineage restriction of a common RGPC^46^,^47^,^48^,^49^. However, the identification of early Cux2-expressing (Cux2^+^) RGPCs, which were reported to be intrinsically specified to generate late-born, upper-layer neurons^38^, calls into question this decades-old model. Our studies show that our established NESCs can specifically produce highly enriched CfuPNs, but Brn2^+^ Cux1^+^ upper layer neurons was not observed in the *in vitro* long-term (pdD70) differentiated cells or in the grafted cells of mouse brain (Figs 3 and 6), indicating that NESCs are probably fate-restricted into cortical deep layer, and human deep-layer projection neurons are most likely generated from lineage-restricted progenitors, similar to two reports in mice^50^,^51^.

Surprisingly, we also find that the fate-restricted NESCs can differentiate into inhibitory cortical interneurons. A number of neurologic and psychiatric disorders, including epilepsy, autism, schizophrenia and possibly Alzheimer’s disease, are thought to result, at least in part, from the dysfunction of cortical interneurons^27^. During development, Calretinin and Parvalbumin subtypes distribute in both E13.5 medial and caudal ganglionic eminences^43^, and Calbindin neurons originate from an anterior extension of the caudal ganglionic eminence^52^. Our study is first time to show that the three subtypes of interneurons can be specified from a NESC, and that CfuPNs and inhibitory interneurons are developed from a common stem cell population in early corticogenesis. Considerable works will be needed to achieve a clear understanding of mechanisms controlling their specifications.

Over other previously developed methods^12^,^21^,^32^, our NESCs have two unique characteristics. The first one is that our NESCs faithfully self-renew. Their cell-cycling parameters, differentiation potentials, marker expression and NT-formation process remain stable for over 30 passes. The other one is that the two processes of individual NESC self-organized into NTs and “NESCs-to-CfuPNs” differentiation are stable, controlled and conserved. These special properties render the power to produce sufficient quantities of functional neurons to facilitate large-scale drug screen, disease modeling and stem cell therapy.

A goal of stem cell field and regeneration medicine is to use stem cell for repairing and replacing damaged tissue and cells *in vivo*. For the purpose, it is crucial to generate neurons with endogenous identities for stem cell therapy. Recent transplantations show PSC-derived cortical progenitors can give rise to CTIP2, CUX1, TBR1 and FOXP2 positive neurons *in vivo*^12^,^34^,^53^. Our NESCs integrate into a wide range of brain regions, and differentiate into TBR1, FOXP2 and SATB2 labeled CfuPNs, and CALRETININ positive interneurons after grafted into newborn mouse brains. Under the continuous observations of five months after transplantation, we fail to observe any tumor formation of grafted cells, suggestive of the safety of NESCs as donor cells for cell therapy. Furthermore, these integrated CfuPNs extensively regenerate axons, and effectively reestablish specific patterns of subcortical projections and synapse structures. These data suggest that NESCs are ideal donor cells to repair the cortex neurologic and psychiatric disorders, such as stroke, cortex injury, epilepsy, autism, schizophrenia and Alzheimer’s disease.

## Materials and Methods

### hESC culture and neuroepthelial stem cell induction

Human cell line BG02 and H9 embryonic stem cells maintained on MEFs as previously described^54^. To induce cortical neuroepthelial stem cells, the ESCs were digested into small clumps for suspension culture on low-cell-adhesion dishes in the medium containing Advance DMEM/F12 (Gibco): Neurobasal media (Gibco) (1:1), 1×B27 (Gibco), and 1% Glutmax (Sigma), 10 ng/mL bFGF (Gibco), 3 μM CHIR99021 (StemRD), 5 μM SB431542 (Cellagen technology), 0.2 μM Compound E (Calbiochem), 0.1 μM LDN193189 (Selleck), and 50 μg/ml Vitamin C (Vc, Sigma). After 6 days, EBs were transferred to 5 μg/ml laminin (Gibco) and poly-ornithine (Sigma, 10 μl/well)-coated plates for attachment culture and the media was switched to CHbFSB + LIF (NESC) culture medium^35^,^36^. The NESC culture medium is composed of Neurobasal media surplus with 1×B27, 1×N2, 1XNEAA (Sigma), 1% Glutmax (Sigma), 3 μM CHIR99021, 5 μM SB431542, 10 ng/ml bFGF, and 1000 U/ml hLIF (Millipore).

### Neuroepthelial stem cell culture and differentiation

To long-term maintain the identity of neuroepthelial stem cells *in vitro*, they were cultured in chemically defined NESC culture medium. 0.05% trypsin (Sigma) was used to digest NESCs when passaging to encourage cell propagation. NESCs were routinely passaged to 1:5 to 1:8 ratios every 4-5 days. For neural tube structure formation, NESCs were continually cultured 8–10 days before passaging.

For differentiation, NESCs were cultured in the media composed of Neurobasal, 1×N2, 1×B27, 1X NEAA and 1% Glutmax. The medium was replaced by fresh medium every 3 days. On Day 7 post-differentiation (pdD7), 10 ng/ml BDNF (Gibco) and 10 ng/ml GDNF (Gibco) were added into the medium to induce terminal differentiation of neurons. To get functional neurons, 2 × 10^3^/cm^2^ mouse astrocytes were added into the differentiated neurons on the one well of 24-well plate at pdD7. Mouse astrocytes were dissociated from new-born mouse cortex. Before used for coculture with neurons, astrocytes were cultured for three passages and no any mouse contaminated neurons were detected in the astrocytes by TUJ1 staining.

### Single cell clone assay

Single NESCs were diluted to approximately 1000 cells/ml, and 1 μl cell suspensions were inoculated on a well of 5 μg/ml laminin and poly-ornithine-coated 96-well plates in NESC culture medium. These wells with one cell were confirmed and these wells with multiple cells are excluded by observation under microscopy 4 hours after inoculations. The media were replaced by refresh media every 2–3 days until Day14 after inoculations. The survival colonies and polarized neural rosette colonies were counted, respectively.

### Immunocytochemistry

Cells were fixed with 4% paraformaldehyde for 15 mins, washed with PBS for three times, treated with 0.2% Triton X-100 (Gibco) for 20 mins, washed with PBS for three times, and incubated in blocking buffer (10% normal donkey serum (Gibco) in PBS) for 30 min at room temperature. The cells were incubated with primary antibody overnight at 4 °C. The cells were washed with PBS including 0.1% Tween 20 and incubated with Alexa 488 or 594 Fluor-conjugated secondary antibodies (Invitrogen: goat anti-rabbit, goat anti-mouse, donkey- anti-goat, donkey anti-chicken, 1: 600) for one hour at RT. The primary antibodies were described in Supplementary (Fig. S3B). Nuclei were visualized by DAPI staining (Sigma-Aldrich).

### RT-PCR

For quantitative RT-PCR, total RNA was isolated from three cultures in the Trizol (Invitrogen) at each differentiation time point (Day 4, 8, 12 and 30), and the cDNA was reserved with M-MLV first strand kit (Invitrogen) according to the manufacturer’s Protocol. The primers used for semi-quantitative PCR and RT- PCR were listed in following (Fig. S3A).

### BrdU incorporation assay

When neural tube structures were formed, bromodeoxyuridine (Sigma) was added to the culture medium to get final 5 μg/ml concentration. An hour later, cells were fixed with 4% PFA, treated by 0.2% Triton X-100 for 20 mins, incubated cells with 1 M HCL for 30 mins at 45 °C, and stained by BrdU antibody.

### Transplantation and Histology

All mouse experiments were performed in Kunming Medical University and the protocol was approved by Kunming Medical University Committee for animal welfare, and the methods were carried out in accordance with the approved guidelines. NESCs were infected by retrovirus containing GFP and performed clonal expansion. Single-derived GFP positive colonies were selected to continually expand. Five GFP positive cell lines were established and one cell line was randomly used for following transplantation.

About 2 μl (2 × 10^5^/μl) of trypsin-dissociated cells in PBS were injected into the ventricles of eleven new born SCID mouse (postnatal 2-5 days). 0.1% food dye was applied to confirm the injection sites. One to three months after transplantation, animals were anesthetized and perfused with 4% paraformaldehyde. Brains were then extracted, post-fixed in 4% PFA (2–3 days) and dehydrated in sucrose solution (10%, 20% and 30%, respectively). The transplanted sites were eventually sectioned on a cryostat microtome at 18 μm after embedding in O.C.T (Sakura-Finetek) and stained with GFP and the neuron or astrocyte antibodies. All antibodies, sources and dilutions are listed in table (Fig. S3B).

### Statistical analysis

All of experiments including immunohohistochemistry were at least performed triplicates. For quantifying neurons, Tuj1 positive cells with a neuronal morphology (a circular, three-dimensional appearance that extended a thin process at least three times longer than their cell body) were quantified as neurons. Quantification was performed on randomly selected 5–8 pictures taken under Leica microscopy using the Leica software package and at least one thousand cells were counted for every experiment. All quantifications were based on at least three independent experiments. Quantification data represented as mean ± standard deviation (s.d) using Microsoft Excel STDEV Function. The significance difference between two samples was evaluated by the unpaired two-sample Student’s *t*-test using Excel software. P < 0.05 was considered as statistical significant differences.

## Additional Information

**How to cite this article**: Zhu, X. *et al*. Efficient Generation of Corticofugal Projection Neurons from Human Embryonic Stem Cells. *Sci. Rep.* **6**, 28572; doi: 10.1038/srep28572 (2016).

## Supplementary Material

Supplementary Information

Supplementary Videos 1

Supplementary Videos 2
