# Supplementary material for: Efficient Generation of Corticofugal Projection Neurons from Human Embryonic Stem Cells
Source: Sci Rep. 2016 Jun 27;6:28572. doi: 10.1038/srep28572 (PMC4921908; doi:10.1038/srep28572)
Supplement: Supplementary Information [file srep28572-s1.pdf]

Efficient Generation of Corticofugal Projection Neurons from Human Embryonic Stem Cells

Xiaoqing Zhu[1,2,3,4], Zongyong Ai[2,4]， Xintian Hu[3*], Tianqing Li[2,4*]

1、School of Life Sciences, University of Science and Technology of China, Hefei, Anhui 230000, PR China

2、Yunnan Key Laboratory of Primate Biomedical Research；Institute of Primate Translational Medicine, Kunming University of Science and Technology, Kunming, 650500, Yunnan, China

3、Key Laboratory of Animal Models and Human Disease Mechanisms of Chinese Academy of Sciences & Yunnan Province, Kunming Institute of Zoology, Kunming, Yunnan, 650223, China

4、Kunming Ennovate Institute of Bioscience，Kunming, 650500, Yunnan, China

*Correspondence: litq@lpbr.cn (T.L.) and xthu@mail.kiz.ac.cn (X.H.).

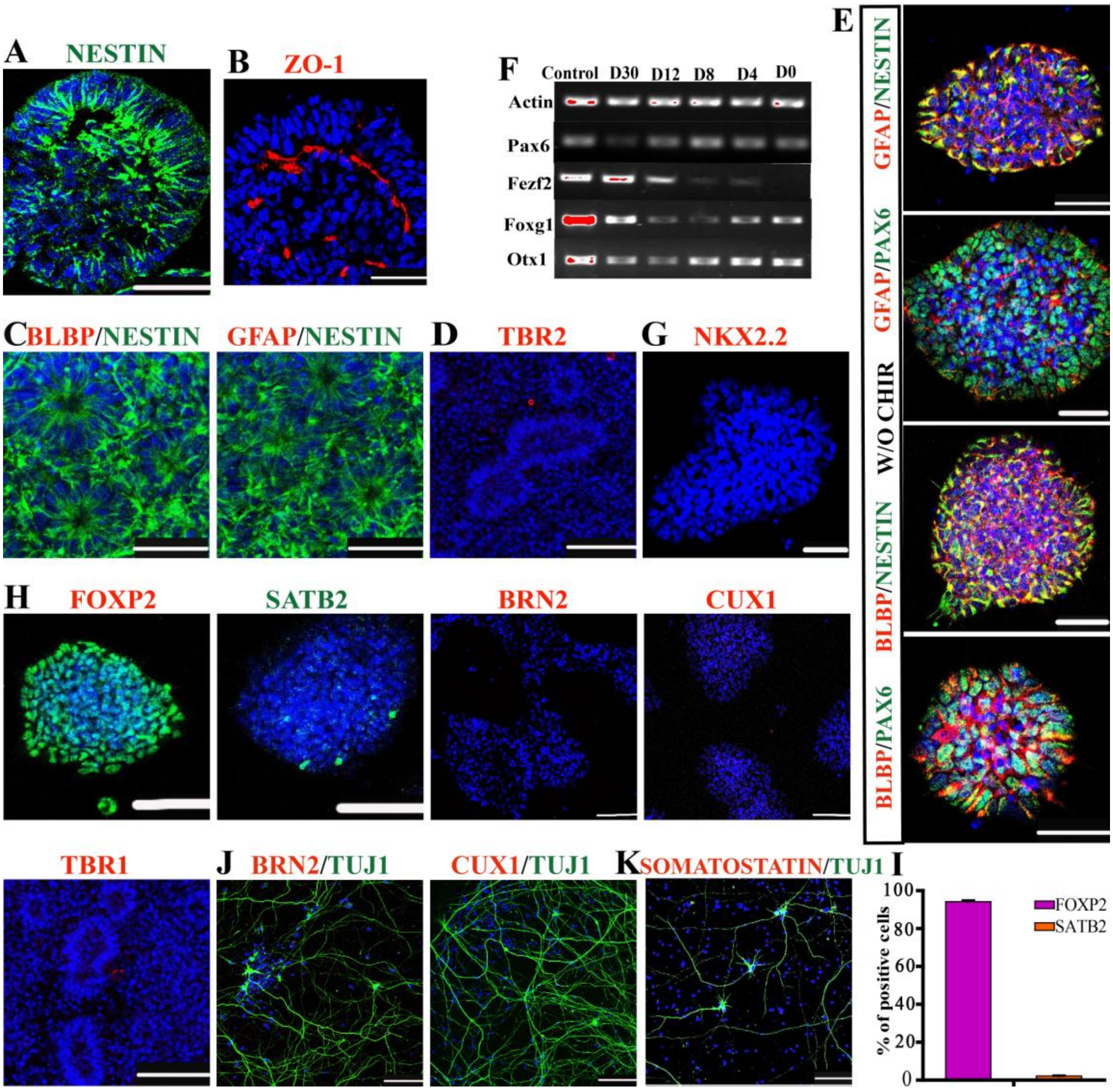

Fig.S1. Neuroepithelial stem cells (NESCs) display the telecephalic identity. (A-B) Immunofluorescence of NESTIN (A) and ZO-1(B) showed the neural body formed a typical two-layer structure. (C-D) NESCs were negative for GFAP and BLBP (two radial glial progenitor cell makers), and Tbr2 (an intermediate cortical progenitor marker). (E) Removal of CHIR99021 in CHbFSB+LIF media resulted in the loss of NT formation and subsequent transition of radial glial progenitor cell (RGPC) from NESCs within 14 days. Transited cells express RGPC markers PAX6 and BLBP, NESTIN and BLBP, PAX6 and GFAP, NESTIN and GFAP. (F) RT-PCR data show that NESCs and its differentiated cells express the transcription factors involved in telecephalic development. (G) NESCs were negative for dorsal marker NKX2.2 in telecephalon. (H) NESCs were positive for FOXP2 (a cortical deep layer marker) along with few SATB2^{+} (a marker of callosal neurons from layers V or upper layer neurons) cells, but not for TBR1 (a marker of cortical deep layer projection neurons) and either BRN2 or Cux1 (two cortical upper

layer markers). (I) Quantification of FOXP2 and SATB2 positive NESCs. Data expressed as mean±s.d (n=3). (J) The neurons derived from NESCs at pdD70 were negative for upper-layer markers BRN2/CUX1. (K) Somatostatin positive neurons were not detected at pdD29. Scale bars: A-B, D, G, J, K, H ,100µm ;C, E, 50µm. Blue: DAPI, nuclear staining.

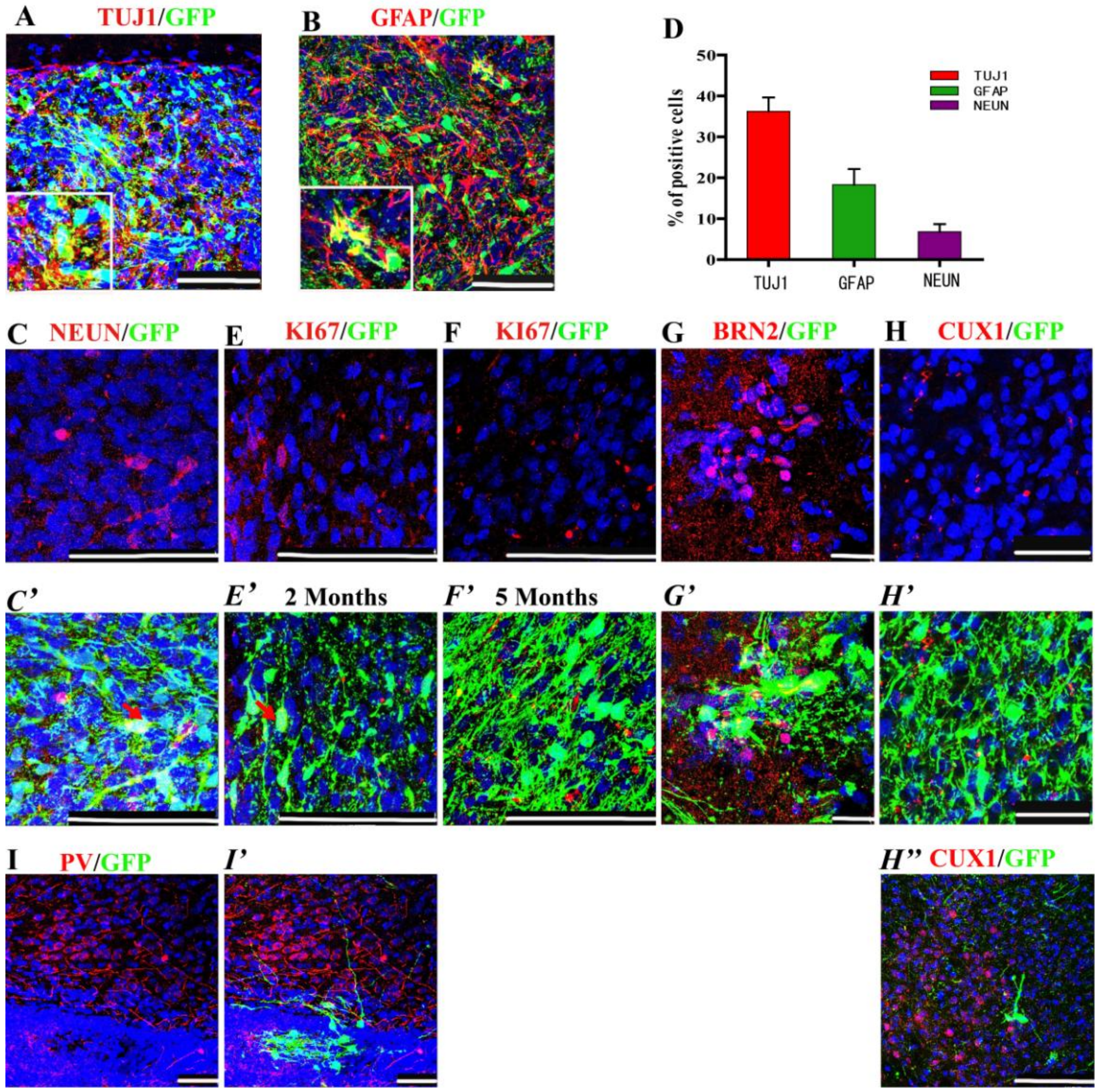

Fig.S2. relative to Figure 5 and 6. Grafted neuroepithelial stem cells (NESCs) differentiated into CfuPNs in mouse brain. (A-B) Immunofluorescence showing TUJ1 positive neurons and GFAP positive astrocytes derived from grafted NESCs. (C) NeuN staining of grafted cells two months after transplantation. Red arrows indicate positive cells. (D) Quantification of TUJ1, GFAP and NeuN positive cells in vivo at two months after transplantation. Data were expressed as mean ±s.d (n=3). (E) A few grafted cells expressed proliferation marker Ki67 two months after transplantation. red arrows indicated positive cells. (F) No any Ki67 cell was detected at the fifth

month post graft. (G-H) No any BRN2 or CUX1 positive upper layer projection neuron was found in grafted three months after transplantation. (I) No any PV (Parvalbumin) positive cell was found in grafted cell. Scale bars: A, B, C-F, I, H,50µm; G, 25µm, H'', 100µm. Blue: DAPI, nuclear staining.

A

Pax6	CCCGTCCATCTTTGCTTG	TCATAACTCCGCCCATTCA
CTIP2	TCACCCACGAAAGGCATCTGT	TGAAGGGCTGCTTGCATGTTG
Otx1	TCTTCGCCAAGACTCGCTAC	CTGCATACACGAGGTGTTGCT
Ngn2	CCGAGGAATCAGAAAGGCTACA	CTCCCGACAAGCACCGCTAT
Foxg1	ACCTCGCTGACACTCCACA	GCACCCGTCAATGACTTCG
Reelin	CAACAGCGCTAGGAGGAAAG	GCCTTCTTCTCGCCTTCTCT
Fezf2	AGCCAGACCGTTCGT	CGGATATGCGTGTTGA
CUX1	ACCATCGGCTTCTTCTACAC	TGGTCAGCGAACTTCTTGG
BRN2	CGGCGGTTTGCTCTATTC	ATGGTGTGGCTCATCGTG
TBR1	TCCCAGTGCCATGTTCCC	AACCCATTTGCCTCCTTGA
SOX5	CCTTCCCATCAAGCACCT	CTCAAAGCCTCTGTCCCA
SATB2	GTGGGCTCAGAGTTAGCAG	TGATGTGGCAATGGAAGAA
β-actin	ACTGGAACGGTGAAGGTGAC	TTTTAGGAGGGCAAGGGAC

B

Antibodies	Company	Cat No.	Dilution
Calretinin	Millpore	AB5054	1:1000
Tbr2	Millpore	AB2283	1:300
NeuN	Millpore	ABN78	1:400
Tuj1	Millpore	MAB1637	1:1000
TH	Millpore	AB152	1:400
CHAT	Millpore	AP144P	1:600
Sox2	Millpore	MAB5603	1:400
Nestin	Millpore	AB5922	1:400
MAP-2	Millpore	AB5622	1:600
Tbr1	Millpore	AB10554	1:200
Synapsin I	Sigma	S193	1:500
Parvalbumin	Sigma	P3088	1:1000
GFAP	Sigma	G6926	1:2000
Brdu	Sigma	B8434	1:300
Brn2	Sigma	SAB2501452	1:400
GABA	Sigma	A2052	1:600
Vglut1	Sigma	V0389	1： 1000
Serotonin	Sigma	S5545	1： 1500
P-Vimentin	MBLInternational	D076-3S	1:200
Satb2	ABCAM	ab 51502	1:200
Foxp2	ABCAM	ab16046	1:250
PSD95	ABCAM	ab2723	1:300
CUX1	SANTACRUZE	SC-7815	1:200
BLBP	ABCAM	ab27171	1:200
S100	ABCAM	ab52642	1:200

Fig.S3. relative to materials and methods. (A)The primers used for semi-quantitative PCR and RT-PCR. (B) The primary antibody list.

Supplemental Videos

Video S1, relative to Figure 5G-b. The 3D reconstruction of SMI312 (a marker of axon) stain demonstrating that the orientations of axon outgrowth were same with that of endogenous neuron axons.

Video S2, relative to Figure 6A. The 3D reconstruction of a representative GFP+ neuron exhibiting a pyramidal morphology in the cortex three months after graft.
